# Does the Duration and Time of Sleep Increase the Risk of Allergic Rhinitis? Results of the 6-Year Nationwide Korea Youth Risk Behavior Web-Based Survey

**DOI:** 10.1371/journal.pone.0072507

**Published:** 2013-08-27

**Authors:** Jeoung A. Kwon, Minjee Lee, Ki-Bong Yoo, Eun-Cheol Park

**Affiliations:** 1 Department of Public Health, Graduate School, Yonsei University, Seoul, Korea; 2 Institute of Health Services Research, Yonsei University College of Medicine, Seoul, Korea; 3 Department of Preventive Medicine, Yonsei University College of Medicine, Seoul, Korea; St. Joseph’s Hospital and Medical Center, United States of America

## Abstract

Allergic rhinitis (AR) is the most common chronic disorder in the pediatric population. Although several studies have investigated the correlation between AR and sleep-related issues, the association between the duration and time of sleep and AR has not been analyzed in long-term national data. This study investigated the relationship between sleep time and duration and AR risk in middle- and high-school students (adolescents aged 12–18). We analyzed national data from the Korea Youth Risk Behavior Web-based Survey by the Korea Centers for Disease Control and Prevention from 2007–2012. The sample size was 274,480, with an average response rate of 96.2%. Multivariate logistic regression analyses were conducted to determine the relationship between sleep and AR risk. Furthermore, to determine the best-fitted model among independent variables such as sleep duration, sleep time, and the combination of sleep duration and sleep time, we used Akaike Information Criteria (AIC) to compare models. A total of 43,337 boys and 41,665 girls reported a diagnosis of AR at baseline. The odds ratio increased with age and with higher education and economic status of the parents. Further, students in mid-sized and large cities had stronger relationships to AR than those in small cities. In both genders, AR was associated with depression and suicidal ideation. In the analysis of sleep duration and sleep time, the odds ratio increased in both genders when sleep duration was <7 hours, and when the time of sleep was later than 24∶00 hours. Our results indicate an association between sleep time and duration and AR. This study is the first to focus on the relationship between sleep duration and time and AR in national survey data collected over 6 years.

## Introduction

The 2008 Allergic Rhinitis and its Impact on Asthma initiative reported that about 500 million individuals suffer from allergic rhinitis (AR) globally [1]. This report showed that AR is the most common chronic disorder in the pediatric population, with up to 40% of children affected [1], [2]. In addition, there is evidence that the prevalence of AR is increasing [1]. According to the Korea Youth Risk Behavior Web-based Survey (KYRBWS-V), the percentage of middle- and high- school students diagnosed with AR gradually increased in Korea from 2007 to 2012, from 24.5% in 2007 to 26.3% in 2008, 27.9% in 2009, 32.2% in 2010, 33.9% in 2011, and 33.9% in 2012 [3]. Therefore, AR should be considered a significant disease among Korean adolescents.

AR has been classified as a major chronic respiratory disease with growing prevalence [1]. Further, a number of studies reported that AR negatively impacts quality of life (QOL) [4]–[9]. The presence of AR symptoms such as nasal congestion, sneezing, nasal itching, and anterior and posterior rhinorrhea is a risk factor for decreasing QOL [4].

Some studies suggest that the impact of AR is more substantive than localized symptoms [4]. Allergy severity has been shown to correlate with reduced work productivity, influencing focus, accuracy, and workload control [10]. Also, AR affects the learning process in children because of daytime fatigue, impaired cognition, and memory [11].

Furthermore, AR has a substantive effect on sleep [12], and can cause fatigue and daytime drowsiness [12], [13]. Nasal congestion due to AR has been shown to negatively influence sleep, causing difficulty falling asleep, hindered sleep, and night awakening [14]–[17]. Interestingly, adolescents (12–17 years) show similar sleep trends of difficulty falling asleep, night awakening, and lack of good sleep [6], [18], [19]. The impact of AR on sleep has an effect on daily function in children as well: children with AR and snoring have been shown to have poorer school performance compared to controls [19]–[21].

In addition, children with AR experience mood problems such as irritability and sadness at school and home [22]. These problems are usually ignored or unnoticed, as children often fail to share them at home or at school [22]. Thus, AR deserves attention from the patient, family, and healthcare professionals [23].

Several studies have investigated the correlation between AR and sleep-related issues. However, the association between the combination of sleep duration and sleep time and AR has not been analyzed in long-term national data. This study is the first to target middle- and high-school boys and girls aged 12–18 to investigate the relationship between sleep duration and sleep time, and AR risk, adjusting for age, current smoking status, current alcohol consumption, maternal educational level, paternal educational level, economic status, type of city, stress, depression, and suicidal ideation.

## Materials and Methods

### Subjects

The nationwide KYRBWS-V survey was conducted from 2007 to 2012 by the Korea Centers for Disease Control and Prevention (KCDCP). The complex research design included multistage sampling, stratification, and clustering [3]. The survey aimed to investigate the prevalence of health-risk behaviors in Korean adolescent students in grades 7–12 [3]. KCDCP has reported all data collection procedures [3]. The questionnaire’s reliability and validity have been assessed by several studies [24], [25].

Participants were given identification numbers and guaranteed anonymity [3]. Survey information was provided by teachers to each student [3]. Each student then completed the web-based survey using the assigned ID number [3]. From 2007 to 2012, the survey included 78,834, 79,099, 76,937, 74,980, 79,202, and 74,186 participants from 798, 796, 800, 800, 800, and 797 middle and high schools, respectively. Response rates were 94.8%, 95.1%, 97.6%, 97.7%, 95.5%, and 96.4%, respectively. A total of 85,002 participants were diagnosed with AR, including Participants of Boys and Girls were 43,337 boys and 41,665 girls. Table 1 lists participant characteristics.

**Table 1:** Characteristics of subjects.

Variables	Boys (n = 137,490)	Girls (n = 136,990)	Total (n = 274,480)	P-value
**Age (years)**	n (%)	n (%)	n (%)	<0.0001
12	7,478(49.0)	7,675(51.0)	15,153(100)	
13	20,052(50.1)	19,870(49.9)	39,922(100)	
14	22,434(50.8)	21,915(49.2)	44,349(100)	
15	24,653(51.2)	23,684(48.8)	48,337(100)	
16	25,243(51.8)	25,016(48.2)	50,259(100)	
17	24,308(50.8)	25,807(49.2)	50,115(100)	
18	13,322(52.8)	13,023(47.2)	26,345(100)	
**Current smoking**				<0.0001
Nonsmoking	115,348(48.3)	128,432(51.7)	243,780(100)	
Smoking	22,142(73.1)	8,558(26.9)	30,700(100)	
**Current alcohol consumption**				<0.0001
Nondrinking	102,946(49.1)	110,292(50.9)	213,238(100)	
Drinking	34,544(58.0)	26,698(42.0)	61,242(100)	
**Educational level of mother**				<0.0001
Middle school or lower	9,680(51.2)	9,877(48.8)	19,557(100)	
High school	76,425(49.8)	79,937(50.2)	156,362(100)	
College or higher	51,385(52.8)	47,176(47.2)	98,561(100)	
**Educational level of father**				<0.0001
Middle school or lower	9,388(52.6)	9,265(47.4)	18,653(100)	
High school	58,751(50.0)	61,237(50.0)	119,988(100)	
College or higher	69,351(51.7)	66,488(48.3)	135,839(100)	
**Economic status**				<0.0001
Very poor	5,629(52.4)	5,565(47.6)	11,194(100)	
Poor	10,802(64.2)	6,260(35.8)	17,062(100)	
Average	36,293(53.8)	31,674(46.2)	67,967(100)	
Wealthy	63,105(48.6)	69,061(51.4)	132,166(100)	
Very wealthy	21,661(48.2)	24,430(51.8)	46,091(100)	
**City type**				<0.0001
Small city	16,134(52.7)	15,938(47.3)	32,072(100)	
Mid-sized city	52,006(50.9)	52,984(49.1)	104,990(100)	
Large city	69,350(51.0)	68,068(49.0)	137,418(100)	
**Stress**				<0.0001
None	3,831(73.8)	1,401(26.2)	5,232(100)	
Low	13,252(40.7)	20,032(59.3)	33,284(100)	
Moderate	37,476(44.5)	48,879(55.5)	86,355(100)	
High	60,658(54.6)	52,522(45.4)	113,180(100)	
Very high	22,273(61.6)	14,156(38.4)	36,429(100)	
**Depression (in 12 months)**				<0.0001
No	95,065(55.0)	80,535(45.0)	175,600(100)	
Yes	42,425(44.0)	56,455(56.0)	98,880(100)	
**Suicidal ideation (in 12 months)**				<0.0001
No	117,054(53.7)	104,974(46.3)	222,028(100)	
Yes	20,436(40.0)	32,016(60.0)	52,452(100)	
**Sleep duration (hours)**				<0.0001
<7	82,837(47.1)	97,202(52.9)	180,039(100)	
≥7	54,653(59.0)	39,788(41.0)	94,441(100)	
**Sleep time (hours)**				<0.0001
Early (<24∶00)	42,414(55.0)	36,381(45.0)	78,795(100)	
Late (≥24∶00)	95,076(49.6)	100,609(50.4)	195,685(100)	
**Sleep duration and sleep time**				<0.0001
≥7 (<24∶00)	38,616(57.2)	30,230(42.8)	68,846(100)	
<7	3,798(38.8)	6,151(61.2)	9,949(100)	
≥7 (≥24∶00)	16,037(63.4)	9,558(36.6)	25,595(100)	
<7	79,039(47.5)	91,051(52.5)	170,090(100)	
**Allergic rhinitis diagnosis**				<0.0001
No	94,153(50.7)	95,325(49.3)	189,478(100)	
Yes	43,337(51.8)	41,665(48.2)	85,002(100)	?

### Dependent Variable

AR history was assessed by the question, “In your life, have you ever been diagnosed with allergic rhinitis by a doctor?” The responses were (1) no and (2) yes.

### Independent Variable

Self-reported sleep duration and sleep time were assessed for each adolescent by asking, “What time did you usually go to bed and wake up last week?” The responses for sleep time were (1) <24∶00 hours and (2) ≥24∶00 hours. The sleep duration was calculated based on reported sleep and awakening times, and the responses were (1) ≥7 hours and (2) <7 hours.

### Covariate Variables

The age range was 12–18 years (middle- and high-school students).

Current smoking status was evaluated by the question, “In the last 30 days, how many days have you smoked more than one cigarette?” Those that responded (1) none were considered nonsmokers. Those with other responses such as (2) 1–2 days per month, (3) 3–5 days per month, (4) 6–9 days per month, (5) 10–19 days per month, (6) 20–29 days per month, and (7) every day were considered smokers.

Current alcohol consumption was evaluated by the question, “In the last 30 days, how many days have you consumed more than one glass of alcohol?” Those responding (1) none were considered non-consumers. Those with other responses such as (2) 1–2 days per month, (3) 3–5 days per month, (4) 6–9 days per month, (5) 10–19 days per month, (6) 20–29 days per month, and (7) every day were considered alcohol consumers.

Parental educational levels were investigated by the questions, “What is your mother’s educational level?” and “What is your father’s educational level?” Response options were (1) middle school or lower, (2) high school, and (3) college or higher.

Economic status was evaluated by the question, “What is your parents’ economic status?” Response options were (1) very wealthy, (2) wealthy, (3) average, (4) poor, and (5) very poor.

City type data were obtained from the students’ address information. The categories were (1) small city, (2) mid-sized city, and (3) large city.

Self-reported stress was evaluated by the question, “How much stress have you been experiencing in your daily life?” The responses were (1) very high, (2) high, (3) moderate, (4) low, and (5) none.

The presence of depression was investigated by the question, “Have you ever experienced a deep sense of sadness or despair in the past 12 months?” The responses were (1) no and (2) yes.

Suicidal ideation was investigated by the question, “Have you ever had suicidal ideation in the past 12 months?” The responses were (1) no and (2) yes.

### Statistical Analysis

Multivariate logistic regression analyses, adjusted for age, current smoking status, current alcohol consumption, maternal educational level, paternal educational level, economic status, type of city, stress, depression, and suicidal ideation, were conducted to assess the relationship between the dependent variable (AR) with sleep duration and sleep time. Furthermore, to determine the best-fitted model among independent variables such as sleep duration, sleep time, and the combination of sleep duration and sleep time, we used Akaike Information Criteria (AIC) to compare models. All data were represented as *N* (%), and odds ratios were calculated by 95% CI. Statistical significance was set at *P*<0.05. Statistical analyses were performed using SAS, version 9.2 (SAS Institute Inc., Cary, NC, US).

## Results

A total of 43,337 (51.8%) boys and 41,665 (48.2%) girls were diagnosed with AR at baseline. Table 1 shows the baseline characteristics of the population.

### Multivariate Logistic Regression Analyses

The results of the multivariate logistic regression analyses of the combination of sleep duration and sleep time and AR are presented in Table 2.

**Table 2:** Multivariate logistic regression analysis*.

Variables	Boys				Girls			
	OR	95% CI		P-value	OR	95% CI		P-value
**Age (years)**								
12	1.00				1.00			
13	1.06	1.00	1.13	0.05	1.06	0.99	1.13	0.10
14	1.10	1.03	1.17	0.01	1.11	1.04	1.18	<.00001
15	1.13	1.06	1.21	<0.0001	1.14	1.07	1.21	<0.0001
16	1.15	1.07	1.23	<0.0001	1.16	1.09	1.24	<0.0001
17	1.20	1.12	1.28	<0.0001	1.28	1.20	1.37	<0.0001
18	1.23	1.14	1.32	<0.0001	1.26	1.17	1.36	<0.0001
**Current smoking**								
Nonsmoking	1.00				1.00			
Smoking	0.92	0.88	0.96	<0.0001	0.95	0.90	1.01	0.11
**Current alcohol consumption**								
Nondrinking	1.00				1.00			
Drinking	0.95	0.92	0.98	<0.0001	0.99	0.95	1.02	0.42
**Educational level of mother**								
Middle school or lower	1.00				1.00			
High school	1.06	1.00	1.13	0.04	1.11	1.05	1.18	<0.0001
College or higher	1.27	1.19	1.36	<0.0001	1.31	1.22	1.39	<0.0001
**Educational level of father**								
Middle school or lower	1.00				1.00			
High school	1.12	1.06	1.19	<0.0001	1.08	1.02	1.15	0.01
College or higher	1.35	1.27	1.44	<0.0001	1.31	1.22	1.40	<0.0001
**Economic status**								
Very poor	1.00				1.00			
Poor	1.10	1.03	1.18	0.01	1.05	0.97	1.13	0.23
Average	1.10	1.02	1.17	0.01	1.04	0.97	1.11	0.32
Wealthy	1.21	1.13	1.30	<0.0001	1.17	1.08	1.26	<.00001
Very wealthy	1.15	1.06	1.24	<0.0001	1.17	1.07	1.28	<0.0001
**City type**								
Small	1.00				1.00			
Mid-sized	1.30	1.23	1.37	<0.0001	1.40	1.32	1.48	<0.0001
Large	1.18	1.12	1.25	<0.0001	1.26	1.19	1.33	<0.0001
**Stress**								
None	1.00				1.00			
Low	1.06	0.98	1.16	0.14	1.03	0.89	1.18	0.70
Moderate	1.21	1.12	1.32	<0.0001	1.12	0.98	1.29	0.10
High	1.35	1.24	1.47	<0.0001	1.27	1.11	1.46	<0.0001
Very high	1.42	1.30	1.56	<0.0001	1.36	1.18	1.56	<0.0001
**Depression (in 12 months)**								
No	1.00				1.00			
Yes	1.06	1.03	1.09	<0.0001	1.08	1.05	1.11	<0.0001
**Suicidal ideation (in 12 months)**								
No	1.00				1.00			
Yes	1.06	1.02	1.11	<0.0001	1.07	1.03	1.11	<0.0001
**Sleep duration and sleep time**								
≥7 (<24∶00)	1.00				1.00			
<7	1.06	0.98	1.16	0.16	0.99	0.92	1.06	0.67
≥7 (≥24∶00)	1.03	0.99	1.08	0.15	1.07	1.01	1.13	0.03
<7	1.12	1.08	1.16	<0.0001	1.15	1.11	1.19	<0.0001

* Adjusting for age, current smoking status, current alcohol consumption, maternal educational level, paternal educational level, economic status, type of city, stress, depression, and suicidal ideation.

Compared with 12-year-old boys, the odds ratios for 13-, 14-, 15-, 16-, 17-, and 18-year-old boys were progressively higher. Compared with 12-year-old girls, the odds ratios for 14-, 15-, 16-, and 17-year-old girls were progressively higher, and that for 18-year-old girls was similar to that for 17-year-old girls.

The association between current smoking status and AR in boys was statistically significant, with an odds ratio of 0.92 (95% CI, 0.88–0.96; *P*<0.0001) for smoking compared with nonsmoking.

The association between current alcohol consumption and AR in boys was statistically significant, with an odds ratio of 0.95 (95% CI, 0.92–0.98; *P*<0.0001) for drinking compared with nondrinking.

In terms of the relationship between maternal education level and AR in boys, the odds ratio was 1.06 (95% CI, 1.00–1.13; *P* = 0.04) for those whose mothers had attended high school, and 1.27 (95% CI, 1.19–1.36; *P*<0.0001) for those with maternal education levels of college or higher compared to middle school or lower. The odds ratios in girls were 1.11 (95% CI, 1.05–1.18; *P*<0.0001) for those whose mothers had attended high school, and 1.31 (95% CI, 1.22–1.39; *P*<0.0001) for those with maternal education levels of college or higher compared to middle school or lower.

With respect to the association between paternal education level and AR in boys, the odds ratios were 1.12 (95% CI, 1.06–1.19; *P*<0.0001) for those whose fathers had attended high school, and 1.35 (95% CI, 1.27–1.44; *P*<0.0001) for those with paternal education levels of college or higher compared to middle school or lower. The odds ratios in girls were 1.08 (95% CI, 1.02–1.15; *P* = 0.01) for those whose fathers had attended high school, and 1.31 (95% CI, 1.22–1.40; *P*<0.0001) for paternal education levels of college or higher compared to middle school or lower.

In terms of the relationship between economic status and AR in boys, the odds ratios were 1.10 (95% CI, 1.03–1.18; *P* = 0.01) for poor, 1.10 (95% CI, 1.02–1.17; *P* = 0.01) for average, 1.21 (95% CI, 1.13–1.30; *P*<0.0001) for wealthy, and 1.15 (95% CI, 1.06–1.24; *P*<0.0001) for very wealthy. In girls, the odds ratios were 1.17 (95% CI, 1.08–1.26; *P*<0.0001) for wealthy, and 1.17 (95% CI, 1.07–1.28; *P*<0.0001) for very wealthy.

The odds ratios for type of city and AR in boys were 1.30 (95% CI, 1.23–1.37; *P*<0.0001) and 1.18 (95% CI, 1.12–1.25; *P*<0.0001) for those living in mid-sized and large cities, respectively, compared to those living in small cities. In girls, the odds ratios were 1.40 (95% CI, 1.32–1.48; *P*<0.0001) and 1.26 (95% CI, 1.19–1.33; *P*<0.0001) for those living in mid-sized and large cities, respectively, compared to those living in small cities.

Among boys, the odds ratios for stress and AR diagnosis progressively increased for moderate, high, and very high stress, compared to those with no stress. In girls, the odds ratios for stress and AR diagnosis progressively increased for those with high and very high levels of stress, compared to those with no stress.

The association between depression and AR was statistically significant in both genders, with odds ratios of 1.06 (95% CI, 1.03–1.09; *P*<0.0001) in boys and 1.08 (95% CI, 1.05–1.11; *P*<0.0001) in girls for depression compared to no depression.

The association between suicidal ideation and AR was statistically significant in both genders, with odds ratios of 1.06 (95% CI, 1.02–1.11; *P*<0.0001) in boys and 1.07 (95% CI, 1.03–1.11; *P*<0.0001) in girls for the presence of suicidal ideation compared to its absence.

In terms of the relationship between the combination of sleep duration and sleep time and AR in boys, the odds ratio was 1.12 (95% CI, 1.08–1.16; *P*<0.0001) for sleep durations of <7 hours after 24∶00 hours compared to sleep durations ≥7 hours before 24∶00 hours. In girls, the odds ratios were 1.07 (95% CI, 1.01–1.13; *P* = 0.03) for sleep durations ≥7 hours after 24∶00 hours and 1.15 (95% CI, 1.11–1.19; *P*<0.0001) for sleep durations <7 hours after 24∶00 hours compared to sleep durations ≥7 hours before 24∶00 hours.

In Table 3, the odds ratios for sleep duration in boys were 1.10 (95% CI, 1.07–1.14; *P*<0.0001), and 1.12 (95% CI, 1.08–1.15; *P*<0.0001) for girls. The odds ratios for sleep time in boys were 1.09 (95% CI, 1.06–1.12; *P*<0.0001), and 1.14 (95% CI, 1.10–1.18; *P*<0.0001) for girls (Table 3).

**Table 3:** Comparison of multivariate logistic regression analysis results and AIC by independent.

Variables	Boys				Girls			
	OR	95% CI		P-value	OR	95% CI		P-value
**Sleep duration (hours)** *								
≥7	1.00				1.00			
<7	1.10	1.07	1.14	<0.0001	1.12	1.08	1.15	<0.0001
AIC	9,237,666.8				8,748,759.8			
**Sleep time (hours)** *								
Early (<24∶00)	1.00				1.00			
Late (≥24∶00)	1.09	1.06	1.12	<0.0001	1.14	1.10	1.18	<0.0001
AIC	9,238,464.6				8,747,707.4			
**Sleep duration and sleep time** *								
≥7 (<24∶00)	1.00				1.00			
<7	1.06	0.98	1.16	0.16	0.99	0.92	1.06	0.67
≥7 (≥24∶00)	1.03	0.99	1.08	0.15	1.07	1.01	1.13	0.03
<7	1.12	1.08	1.16	<0.0001	1.15	1.11	1.19	<0.0001
AIC	9,237,440.6				8,747,197.4			

* Adjusting for age, current smoking status, current alcohol consumption, maternal educational level, paternal educational level, economic status, type of city, stress, depression, and suicidal ideation.

### Model Fit Comparison Analyses

To investigate the model fit among three independent variables such as sleep duration, sleep time, and the combination of sleep duration and sleep time, AIC was assessed. The AIC value for sleep duration was 9,237,666.8 in boys and 8,748,759.8 in girls, that for sleep time was 9,238,464.6 in boys and 8,747,707.4 in girls, and that for the combination of both sleep duration and sleep time was 9,237,440.6 in boys and 8,747,197.4 in girls. The best-fitted model with the smallest AIC was one using the combination of sleep duration and sleep time (Table 3).

## Discussion

AR is a common disease that affects about 40% of the pediatric population [4]. This study focused on the adolescent population aged 12–18 years. Adolescence is accompanied by rapid and varied changes in physical growth, sexual maturity, hormone levels, and psychological issues [26], [27]. Moreover, Korean adolescents suffer from lack of sleep hours due to study load. Several studies have investigated the relationship between sleep issues and AR. However, this study is the first to investigate sleep duration and sleep time simultaneously as a variable.

In multivariate regression analysis, the odds ratios in boys aged 13–18 years and in girls aged 14–18 years were statistically significant compared to those aged 12 years. The associations between sleep duration and sleep time, and AR, increased with age in both genders. This trend indicates the prevalence of AR as a function of age in Korea.

In boys, the odds ratios for both smoking and alcohol consumption group were lower than in the non-consuming groups. These results can be interpreted that boys with AR were more aware of their health than those without AR. This shows the effect of disease on healthy behavior.

A stronger relationship with AR was seen in both genders with higher parental education levels. Moreover, the odds ratios between economic status and AR also increased with higher parental socioeconomic levels. A positive relationship was observed with highly-educated parents and higher parental socioeconomic level. Children of highly-educated parents and those of high economic status were more likely to be diagnosed with AR than children of less-educated parents and those of low economic status [28].

In addition, children who resided in large cities and mid-sized cities were more likely to have been diagnosed with AR than those living in small cities. This result implies that large cities and mid-sized cities may have more sources of pollution that stimulates the autoimmune system.

The results of this study showed that AR was positively associated with stress levels in both genders. The relationship between depression and AR was statistically significant. Furthermore, the relationship between suicidal ideation and AR was statistically significant. These three independent variables are associated with mental status and are closely related. This is due to the stress-related hypothalamic-pituitary-adrenal axis reaction to psychological stressors and the cortisol response after psychological stress [29]. The causal factors of stress can be disease symptoms or other substantive impacts such as sleep disturbance due to these symptoms. Many studies have investigated the relationship among stressors, depression, and suicidal ideation [30]–[36]. This study proved the association among the three variables of stress, depression, and suicidal ideation.

With respect to the combination of sleep duration and sleep time, the odds ratio was significantly increased in girls with sleep durations of more than 7 hours after 24∶00 hours compared to sleep before 24∶00 hours. Moreover, the odds ratio was increased in both genders with sleep durations of less than 7 hours after 24∶00 hours compared to before 24∶00 hours. However, the odds ratio was not statistically significant for sleep durations of less than 7 hours before 24∶00 hours. In addition, the odds ratio was significantly higher for sleep durations less than 7 hours compared to more than 7 hours in boys who slept before 24∶00 hours. Furthermore, in both genders, the odds ratio was significantly increased for sleep durations less than 7 hours compared to more than 7 hours when the sleep time was later than 24∶00 hours. From this result, not only sleep duration, but also sleep time, influences AR risk. Therefore, to have sufficient sleep, parents and adolescents need to consider both sleep duration and sleep time at the same time. Moreover, to improve sleep quality, sleep interruption factors such as AR symptoms have to be treated carefully.

To our knowledge, this is the first study to examine the association between sleep and AR considering both sleep duration and sleep time using multi-year national survey data (2007–2012). The survey was large in scale and relied on a complex design including multistage sampling, stratification, and clustering [3]. The reliability and validity of the questionnaire data have been assessed by previous studies [24], [25]. Additionally, the average response rate from 2007 to 2012 to the survey was 96.2%, representing a majority of Korean adolescents.

However, this study has limitations. First, this study design was cross-sectional; therefore, it examined the interrelationship between sleep time and AR rather than issues of causality. Thus, the results of this study should be interpreted cautiously. Additionally, some confounding variables, such as AR severity, may not have been included in this study. Moreover, the data on the socioeconomic status of the respondents may not be accurate because students rather than parents provided this information. Because we relied on self-reports for measurements of stress, these responses may be subjective.

Even though this study has some limitations, it is the first to simultaneously focus on sleep duration and sleep time in national survey data for 6 years. Future studies should include other factors that can affect the relationship between sleep and AR.
